# The effects of ultrasound-guided serratus anterior plane block on intraoperative opioid consumption and hemodynamic stability during breast surgery: A randomized controlled study

**DOI:** 10.1097/MD.0000000000030290

**Published:** 2022-09-02

**Authors:** Çağdaş Baytar, Bahar Aktaş, Bengü Gülhan Aydin, Özcan Pişkin, Güldeniz Karadeniz Çakmak, Hilal Ayoğlu

**Affiliations:** a Department of Anesthesiology and Reanimation, Faculty of Medicine, Zonguldak Bülent Ecevit University, Zonguldak, Turkey; b Department of Anesthesiology and Reanimation, Çaycuma State Hospital, Zonguldak, Turkey; c Department of General Surgery, Faculty of Medicine, Zonguldak Bülent Ecevit University, Zonguldak, Turkey.

**Keywords:** breast cancer surgery, emergence time, hemodynamics, opioids, serratus anterior plane block, ultrasound-guided

## Abstract

To determine effect of ultrasound-guided serratus anterior plane block (SAPB) on intraoperative opioid consumption in patients undergoing oncoplastic breast surgery under general anesthesia.

**Methods:**

This study was conducted as a prospective, randomized controlled trial. Forty-four patients enrolled, aged 18 to 75 years with American Society of Anesthesiologists physical status I to III, undergoing elective oncoplastic breast surgery. Patients were randomly allocated to receive SAPB with 20 mL of 0.25% bupivacaine + general anesthesia (group SAPB) or only general anesthesia (group control). The primary outcome was assessing the effect of SAPB on intraoperative remifentanil consumption. Patients were assessed for emergence time, hemodynamic parameters, doses of rescue drugs used to control hemodynamic parameters, and duration of stay in the recovery room.

**Results:**

Preoperative SAPB with 0.25% bupivacaine reduced intraoperative opioid consumption (851.2 ± 423.5 vs 1409.7 ± 756.1 µg, *P* = .019). Emergence time was significantly shorter in group SAPB (6.19 ± 1.90 minutes) compared to group control (9.50 ± 2.39 minutes; *P* < .001). There were no significant differences in the doses of rescue drugs used for systolic blood pressure and heart rate between the groups.

**Conclusions:**

Preoperative SAPB with bupivacaine reduced intraoperative opioid consumption and shortened emergence time and duration of stay in the recovery unit, and hemodynamic stability was maintained without block-related complications.

## 1. Introduction

Oncoplastic breast surgery is among the most frequently performed surgeries for breast cancer.^[1]^ It has been recommended that surgical pain should be managed using a multimodal analgesic regimen during the perioperative period.^[2]^

Opioids are typically used to control perioperative pain. However, opioids have many side effects, such as nausea, vomiting, itching, constipation, respiratory depression, and urinary retention.^[3]^ In addition, intraoperative high-dose opioid consumption causes an increased risk of opioid-induced hyperalgesia and postoperative nausea and it is associated with a prolonged recovery time.^[4]^ Furthermore, studies have shown that short-acting opioids consumed intraoperatively in fact increase postoperative pain scores if consumed at high doses.^[5–7]^ In light of the limitations of opioids in perioperative pain management, a committee established by the American Society of Breast Surgeons recently reached a consensus that opioid use should be reduced in patients undergoing breast surgery.^[2]^

Given the limitations of opioids in pain management, a multimodal approach is beneficial. One technique that may be considered is regional analgesia. Regional analgesia techniques, besides being part of the pain management of patients, also contribute to the provision of hemodynamic stability during the intraoperative period.^[8]^ Serratus anterior plane block (SAPB) is a simple, effective, and safe regional analgesia technique applied to the thoracic and chest walls with few side effects.^[9,10]^ In recent years, it is a popular block that has been increasingly used in pain management in breast surgery.^[11]^

In this study, we aimed to investigate the effect of preoperative SAPB on intraoperative remifentanil consumption, hemodynamic parameters, emergence time, and duration of stay in the recovery unit in patients undergoing oncoplastic breast surgery under general anesthesia.

## 2. Materials and Methods

The present study was conducted as a prospective, randomized, and controlled study after the approval of the Ethics Committee (Ref No 2020/03-12, ClinicalTrials.gov identifier: NCT04824833). The CONSORT guidelines were followed for this study.

Forty-four female patients, American Society of Anesthesiologists physical status I to III, aged 18 to 75 years, who underwent elective oncoplastic breast surgery under general anesthesia were included in the study. Exclusion criteria were bilateral surgery, a history of allergy to the local anesthetics used, infection in the area to be blocked, coagulopathy, and patient’s refusal to participate in the study. Four patients were excluded from the study (3 of them did not want to participate in the study; 1 patient’s surgery was bilateral). Forty patients were divided into 2 groups in a 1:1 ratio using the closed envelope method after the randomization list was prepared through the computer program: group SAPB (general anesthesia + SAPB, n = 20) and group control (general anesthesia, n = 20).

All SAPB procedures were performed by the same anesthesiologist (Ç.B.). After standard monitoring, 1 mg of midazolam was administered to patients for sedation. While patients were in the supine position, the lateral chest wall to be blocked was sterilized. A high-frequency linear ultrasound probe (MyLab 30 Gold Cardiovascular; Esaote, Florence, Italy) wrapped in a sterile sheath was placed on the mid-axillary line at the 5th rib. The latissimus dorsi muscle and serratus anterior muscle (SAM) were recognized above the ribs, and a 22-gauge 80 mm block needle was advanced between the SAM and 5th rib. After confirming the application site by hydrodissection, 20 mL of 0.25% bupivacaine was administered. The success of the block was evaluated by testing the loss of sensation using a blunt needle. Subsequently, patients were transferred to the operating room. All surgeries were performed by the same surgeon (G.K.Ç.).

General anesthesia was applied in both groups after train-of-four (TOF; TOF-Watch SX), bispectral index monitoring (BIS), and Pleth Variability Index (PVI; Masimo-Radical-7 ™ Pulse CO-Oximeter®) monitoring, in addition to standard monitoring. preanesthesia blood pressure and heart rate (HR) values were recorded. Propofol (2 mg/kg), 1 µg/kg bolus remifentanil (within 1 minute), and rocuronium (0.6 mg/kg) were administered. Patients were intubated after confirmation of maximum neuromuscular blockade (NMB) using TOF. After intubation, blood pressure and HR were measured 3 times at 1-minute intervals and recorded. The basal systolic blood pressure (SBP) and HR were determined by taking the average of these 3 values. Anesthesia was maintained with propofol infusion for a BIS value of 40 to 50, and remifentanil infusion (0.2–1 µg/kg/min) for SBP and HR within 70% to 130% of the baselines. When the TOF count reached 2 or higher during surgery, 0.15 mg/kg rocuronium was administered to the patient. During the operation, the patient’s fluid therapy was adjusted to a PVI of 14% to 16%. SBP and HR were recorded immediately after incision and subsequently at 5, 15, 30, 60, 90, and 120 minutes during surgery.

A 30% increase from baseline HR and SBP values after intubation was taken as evidence of pain. If an increase equal to, or above this value, was observed, the remifentanil dose was increased. Conversely, if a reduction of 30% or more from the baseline was observed, the dose of remifenanil was reduced. The remifentanil dose was increased to a maximum of 1 µg/kg/min. Despite these dose adjustments, if the SBP and HR values could not be controlled within the specified range, atropine, esmolol, perlinganite, and ephedrine were administered as rescue drugs and recorded. Propofol infusion was terminated with the last subcutaneous suture. After skin suture was completed, remifentanil infusion was terminated. The time between the last suture and extubation was defined as emergence time. IV Paracetamol (10 mg/kg) was administered to all patients 15 minutes before the end of surgery. At the end of surgery, when the TOF count reached 2, an appropriate dose of atropine and neostigmine was administered. After regaining consciousness and a TOF ratio >0.9, the patient was extubated. The patient was transferred to the recovery unit when their BIS value exceeded 80, and they showed effective spontaneous breathing and an ability to follow simple instructions. Patients with a modified Aldrete score of ≥ 9 were transported to the clinic, and the duration of stay in the recovery room was recorded.

The primary outcome of our study was the required dosage of intraoperative remifentanil consumption. Secondary outcomes were emergence time, hemodynamic parameters, doses of rescue drugs used to control hemodynamic parameters, and duration of stay in the recovery room.

## 3. Statistical analysis

A power analysis was conducted prior to the study. In the sample size analysis performed using the reference power = 0.99 and confidence interval = 0.98 (group 1: 1047 ± 390, group 2: 519 ± 246,^[12]^ the minimum number of patients to be reached was found to be 38. Considering a 10% to 15% attrition rate, the required sample size was determined to be 44. SPSS 22 Windows (Statistical Package for Social Sciences, Armonk, NY) was used to analyze the data. Descriptive data are shown as the mean ± standard deviation. The Kolmogorov–Smirnov test was used as a test for normality of distribution. For comparative data, Mann–Whitney *U* test, paired *t* test, and *t* test were used in the analysis of data for nonnormal distribution. Nonparametric data were analyzed using the chi-square test. Statistical significance was set at *P* < .05.

## 4. Results

Forty of the 44 patients who underwent oncoplastic breast surgery were included and evaluated statistically (Follow Diagram). The patients’ demographic data, duration of anesthesia, and surgery were statistically similar between the groups (Table 1). There were no differences between the groups in terms of SBP and HR before and after intubation, or during surgery (Table 2).

**Table 1:** Demographic and clinical data.

	Group SAPB (n = 20)	Group control (n = 20)	*P*
Age (yr)	49.55 ± 9.17	53.75 ± 10.78	.167
Height(cm)	162.1 ± 3.6	163.0 ± 4.4	.642
Weight (kg)	77.3 ± 13.8	76.7 ± 12.9	.787
BMI (kg/m^2^)	29.41 ± 5.15	28.82 ± 4.39	.665
ASA (I/II/III)	0/17/3	1/15/4	
Anesthesia time(min)	147.3 ± 55.9	152.5 ± 58.1	.818
Surgery time(min)	128.9 ± 54.7	130.7 ± 57.2	.978

Data presented as mean ± standard deviation.

ASA = American Society of Anesthesiologists, BMI = body mass index, SAPB = serratus anterior plane block.

**Table 2:** Hemodynamic data.

	Group SAPB (n = 20)	Group control (n = 20)	*P*
SBP (before anesthesia)	137.6 ± 14.7	136.9 ± 26.5	.355
SBP (after intubation)	130.3 ± 27.4	137.7 ± 27.4	.403
HR (before anesthesia)	84.2 ± 12.0	85.4 ± 14.4	.946
HR (after intubation)	86.1 ± 17.2	86.1 ± 16.3	1

Data presented as mean ± standard deviation.

HR = heart rate, SAPB = serratus anterior plane block, SBP = systolic blood pressure.

Intraoperative remifentanil consumption was significantly lower in group SAPB (851.2 ± .423.5 µg) than in group control (1409.7 ± 756.1 µg; *P* = .019; Table 3).

**Table 3:** Intraoperative remifentanil consumption, emergence time, and duration of stay in recovery unit.

	Group SAPB (n = 20)	Group control (n = 20)	*P*
Intraoperative Remifentanil dose (µg)	851.2 ± 423.5	1409.7 ± 756.1	.019*
Emergence time (min)	6.19 ± 1.90	9.50 ± 2.39	<.001*
Duration of stay in recovery unit (min)	22.5 ± 5.7	30.2 ± 6.3	<.001*

Data presented as mean ± standard deviation.

SAPB = serratus anterior plane block

* *P* < .05.

**Figure 1.**
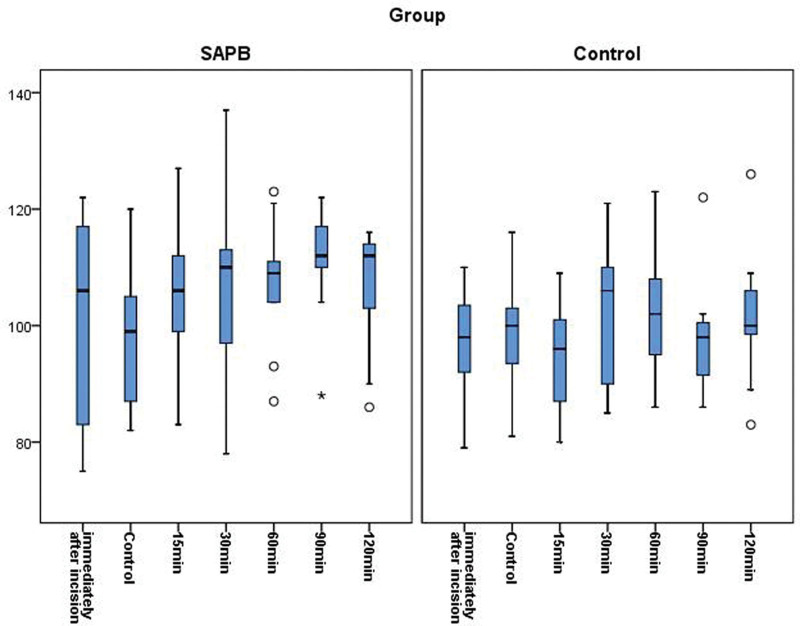
Time course of systolic blood pressure.

**Figure 2.**
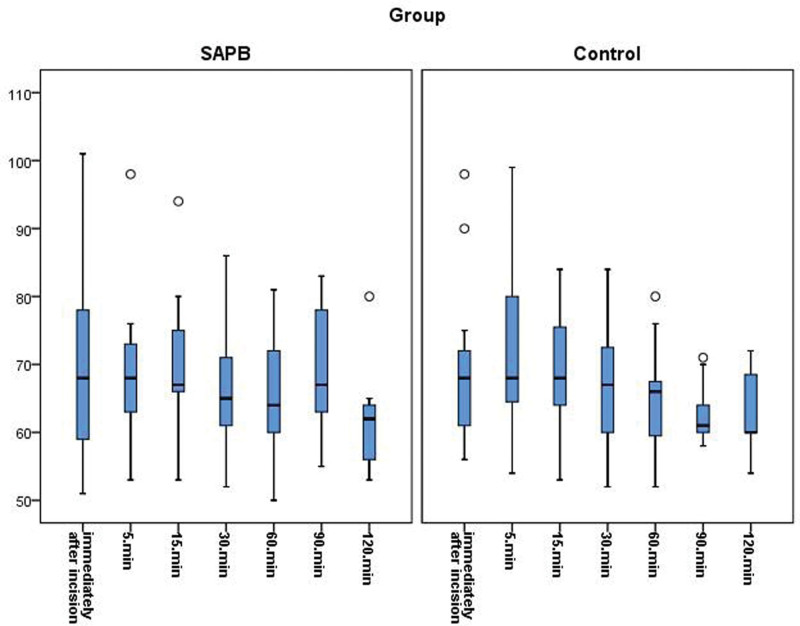
Time course of heart rate.

The emergence time was significantly shorter in group SAPB (6.19 ± 1.90 minutes) than in group control (9.50 ± 2.39 minutes; *P* < .001; Table 3). There were no significant differences between the groups at any time point with regard to SBP and HR (Figs. 1 and 2).

There were no significant differences in the doses of rescue drugs used for SBP or HR between the groups. No patient in either group received perlinganit or esmolol. In group SAPB ephedrine was administered to 2 patients and atropine was administered to 3 patients. In the control group, only 1 patient was administered ephedrine. The duration of stay in the recovery unit was significantly shorter in group SAPB (22.5 ± 5.7 minutes) than in group control (30.2 ± 6.3 minutes; *P* ≤ .001; Table 3). No complications occurred during or after block application.

## 5. Discussion

In the present study, we demonstrated that ultrasound-guided SAPB reduced intraoperative opioid consumption in patients undergoing oncoplastic breast surgery under general anesthesia. Additionally, SAPB reduced the emergence time and duration of stay in the recovery room without instability of hemodynamic parameters during the intraoperative period.

One of the most important components of balanced anesthesia is the management of nociception, and opioid administration is generally preferred for intraoperative pain control.^[13]^ However, opioids have many side effects, such as sedation, dizziness, nausea, vomiting, constipation, tolerance, and respiratory depression. Other known side effects include hyperalgesia, muscle rigidity, and delayed gastric emptying. Intolerance to some of these side effects does not develop and may be severe enough to cause treatment interruption.^[14]^ Physical dependence is also of great concern. It is well known that opioid use disorder and opioid addiction have reached epidemic proportions in the United States and the world, with millions of people suffering from these conditions.^[15]^

Neuroadaptation to opioid use causes a phenomenon called opioid-induced hyperalgesia. In this clinical syndrome, opioid requirement and pain scores increase in the postoperative period because of high-dose opioid consumption during the intraoperative period.^[16]^ In a meta-analysis investigating the clinical results of intraoperative opioid consumption doses, opioid-induced hyperalgesia was more prominent with intraoperative high use of short-acting opioids such as fentanyl and remifentanil.^[17]^ In light of these, the aim is to become distanced from opioid use as much as possible in the perioperative period in anesthesia practice. In this study, we investigated the effect of SAPB on intraoperative remifentanil consumption in patients undergoing oncoplastic breast surgery.

Blanco et al^[18]^ described that SAPB can be applied both deep and superficially by providing long-term regional anesthesia in surgeries performed on the chest wall in 2013. SAPB is applied safely, easily, and rapidly as part of multimodal analgesia in thoracic, cardiac, and breast surgery.^[9]^ In deep SAPB, a local anesthetic is applied between the SAM and the 5th rib. The lateral cutaneous branches of the intercostal nerves are blocked, and sensory loss occurs between T2 and T8 dermatomes.^[18]^ Edwards et al^[19]^ reported that deep SAPB applied for postoperative analgesia in patients undergoing mastectomy reduced opioid consumption by 30% during 24 hours postoperatively. Bakeer et al^[20]^ showed that deep SAPB in patients undergoing modified radical mastectomy reduced intraoperative fentanyl requirement and postoperative morphine consumption. In the present study, we determined that preoperatively administered deep SAPB significantly reduced intraoperative remifentanil consumption compared to that in the group that received only general anesthesia.

Alternatives such as thoracic epidural analgesia and paravertebral block are available for pain management in patients undergoing breast surgery.^[21,22]^ However, these blocks lead to hemodynamic changes such as hypotension and bradycardia caused by sympathetic blockade.^[23]^ SAPB is a peripheral block that does not cause a sympathetic blockade. In this study, there were no significant differences between the groups in terms of SBP and HR during surgery. Furthermore, there were no significant differences in the doses of rescue drugs used for SBP or HR between the groups. Stable hemodynamics were achieved by consuming less remifentanil, with the analgesic effect of SAPB applied before the incision.

Opioids may also contribute to a prolonged emergence time due to sedation and respiratory depression. Synergy with other anesthetic agents may also enhance the sedative effects of opioids. The use of opioids in a multimodal analgesic regimen also contributes to an increased emergence time due to hypercapnia following respiratory depression.^[24]^ However, insufficient pain control is associated with inadequate spontaneous respiration and can delay extubation.^[25]^ In our study, the emergence time was significantly shorter in the SAPB group than in the control group. We consider that this is due to the fact that the SAPB provides effective analgesia and reduces opioid consumption.

Opioids have been shown to prolong the length of stay in the recovery room.^[4]^ In our study, regarding the duration of stay in the recovery room, there were significant differences between the SAPB (22.5 minutes) and control groups (30.2 minutes).

Our study has some limitations. We did not use a tool as an objective measure of pain based on heart rate variability like Surgical Pleth Index and Analgesia Nociception Index. We did not evaluate the pain scores and opioid consumption of the patients during the postoperative period. However, several studies have reported both visual analog scale scores and opioid consumption in the postoperative period in patients undergoing breast surgery.^[19,20]^ Another limitation was that we did not follow the effect of opioid consumption on nausea and vomiting in the recovery room.

In conclusion, we demonstrated that SAPB reduced intraoperative remifentanil consumption in patients undergoing oncoplastic breast surgery under general anesthesia. In addition, SAPB shortened the emergence time and duration of stay in the recovery unit, and hemodynamic stability was maintained without block-related complications. Within the framework of our results, we recommend application of preoperative SAPB in patients who undergo oncoplastic breast surgery under general anesthesia.
